# Cucurbitacin IIb Exhibits Anti-Inflammatory Activity through Modulating Multiple Cellular Behaviors of Mouse Lymphocytes

**DOI:** 10.1371/journal.pone.0089751

**Published:** 2014-02-25

**Authors:** Yao Wang, Gao-Xiang Zhao, Li-Hui Xu, Kun-Peng Liu, Hao Pan, Jian He, Ji-Ye Cai, Dong-Yun Ouyang, Xian-Hui He

**Affiliations:** 1 Department of Immunobiology, College of Life Science and Technology, Jinan University, Guangzhou, China; 2 Department of Cell Biology, College of Life Science and Technology, Jinan University, Guangzhou, China; 3 Department of Chemistry, College of Life Science and Technology, Jinan University, Guangzhou, China; Temple University School of Medicine, United States of America

## Abstract

Cucurbitacin IIb (CuIIb) is one of the major active compounds in Hemsleyadine tablets which have been used for clinical treatment of bacillary dysentery, enteritis and acute tonsilitis. However, its action mechanism has not been completely understood. This study aimed to explore the anti-inflammatory activity of CuIIb and its underlying mechanism in mitogen-activated lymphocytes isolated from mouse mesenteric lymph nodes. The results showed that CuIIb inhibited the proliferation of concanavalin A (Con A)-activated lymphocytes in a time- and dose-dependent manner. CuIIb treatment arrested their cell cycle in S and G_2_/M phases probably due to the disruption of the actin cytoskeleton and the modulation of p27^Kip1^ and cyclin levels. Moreover, the surface expression of activation markers CD69 and CD25 on Con A-activated CD3^+^ T lymphocytes was suppressed by CuIIb treatment. Both Con A- and phorbol ester plus ionomycin-induced expression of TNF-α, IFN-γ and IL-6 proteins was attenuated upon exposure to CuIIb. Mechanistically, CuIIb treatment suppressed the phosphorylation of JNK and Erk1/2 but not p38 in Con A-activated lymphocytes. Although CuIIb unexpectedly enhanced the phosphorylation of IκB and NF-κB (p65), it blocked the nuclear translocation of NF-κB (p65). In support of this, CuIIb significantly decreased the mRNA levels of *IκBα* and *TNF-α*, two target genes of NF-κB, in Con A-activated lymphocytes. In addition, CuIIb downregulated Con A-induced STAT3 phosphorylation and increased cell apoptosis. Collectively, these results suggest that CuIIb exhibits its anti-inflammatory activity through modulating multiple cellular behaviors and signaling pathways, leading to the suppression of the adaptive immune response.

## Introduction

**Figure 1:**
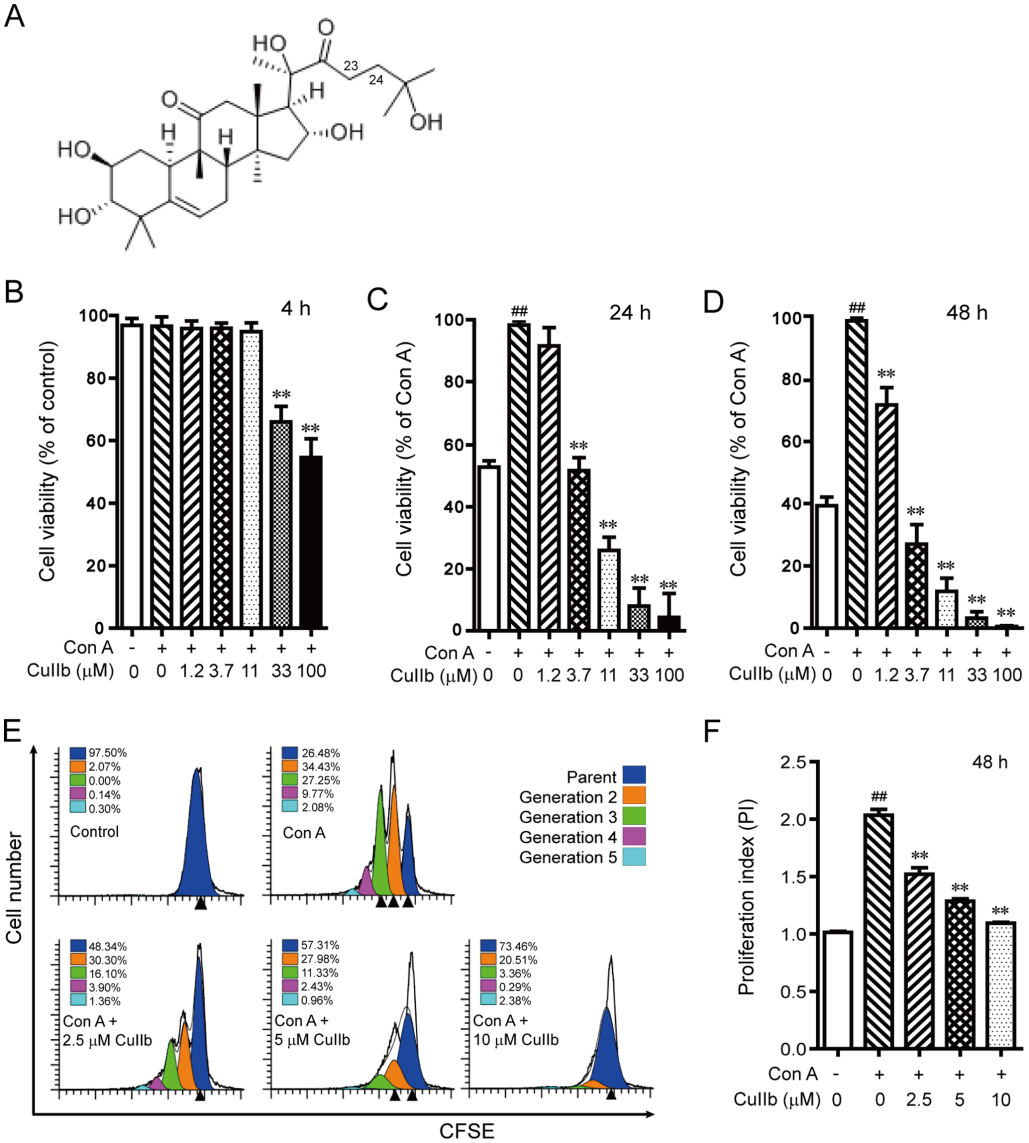
The chemical structure of cucurbitacin IIb (CuIIb) (A) and its effect on the proliferation of Con A-stimulated lymphocytes. Mouse lymphocytes were incubated with different concentrations of CuIIb for 4 h, 24 h or 48 h, respectively and analyzed with WST-1 assay (B, C and D). Cell division was also measured by CFSE staining assay (E) and the proliferation indexes of CFSE-labeled cells are presented in (F). Data are analyzed with ModFit software. One representative data of three independent experiments with similar results are shown as mean ± SD (n = 3). ##*P*<0.01 versus control group; **P<0.01 versus Con A group.

Cucurbitacins belong to a large family of triterpenoid compounds found in Cucurbitaceae plants and they possess a wide spectrum of biological and pharmacological activities [1], [2], [3], [4], [5]. As a member of cucurbitacin family, cucurbitacin IIb [6] (also known as hemslecin B or 23,24-dihydrocucurbitacin F; **Figure 1A**) is isolated from the medicinal plant *Hemsleya amabilis* (Cucurbitaceae), which has long been used as a folk remedy for bacillary dysentery and enteritis. The root of *Hemsleya amabilis* contains two closely related cucurbitacins: cucurbitacin IIa and IIb (CuIIb); Hemsleyadine tablets made of *Hemsleya amabilis* root extract, which contain both cucurbitacin IIa and IIb, have also been shown effective for bacillary dysentery, enteritis and acute tonsillitis [7]. These clinical observations suggest that cucurbitacin IIa and IIb have anti-inflammatory properties. However, the action mechanism underlying such anti-inflammatory effects is largely unknown, although our previous study has shown that cucurbitacin IIa induces apoptosis and autophagy in lipopolysaccharide-activated RAW 264.7 macrophages indicating a potential action on the innate immune response [8].

T lymphocytes play a critical role in shaping both the innate and adaptive immune responses through either direct or indirect interaction with other immune cells, secreting a variety of cytokines including interferon (IFN)-γ, tumor necrosis factor (TNF)-α and interleukin (IL)-6 [9], [10]. Upon antigen recognition, a naïve T lymphocyte activates several critical signaling pathways, such as the mitogen-activate protein kinases (MAPKs) and NF-κB pathways [11], [12] and undergoes profoundly changes including activation, proliferation, and cytokine expression, leading to a robust clonal expansion. All these processes may be pharmacologically manipulated, thus representing important targets for therapeutic invention against inflammatory diseases [13]. In the course of inflammation-related diseases, enteritis for example, both innate and adaptive immune cells including T lymphocytes infiltrate the inflamed tissues of the intestine [14], suggesting that the lymphocytes are potential targets of anti-inflammatory drugs like CuIIb. Thus, it is of interest to explore the effects of CuIIb on the cellular behaviors of lymphocytes in response to mitogenic stimulation.

In this study, we adopted *in vitro* activated mouse lymphocytes as a cellular model to explore the anti-inflammatory activity of CuIIb and the underlying action mechanism. Our results showed that CuIIb inhibited the activation and proliferation of Con A-activated lymphocytes as well as their inflammatory cytokine expression. Mechanistically, such effects of CuIIb were likely mediated by blocking nuclear translocation of transcription factor NF-κB and by downregulating JNK and Erk1/2 signaling in the activated lymphocytes. Thus, CuIIb may exhibit anti-inflammatory activity by the suppression of adaptive immune responses of lymphocytes.

## Materials and methods

### Animals and reagents

Female BALB/c mice, 6 − 8 weeks of age, were supplied by the Experimental Animal Center of Southern Medical University (Guangzhou, China). Animal experiments were performed in accordance with the Guidelines for the Care and Use of Laboratory Animals of Jinan University. The protocol was approved by the Committee on the Ethics of Animal Experiments of Jinan University (No. JNU20120305). Cucurbitacin IIb (purity: 98.0 %) was obtained from Shanghai Shunbo Biotech Co. (Shanghai, China). Phorbol 12, 13-dibutyrate (PDB), ionomycin (Ion), monensin, concanavalin A (Con A), propidium iodide (PI) and dimethyl sulfoxide (DMSO) were purchased from Sigma-Aldrich (St. Louis, MO, USA). CuIIb was dissolved in DMSO at 20 mM, and stored at −20°C. Diluted working solution was prepared freshly before each experiment. 6-(N-Succinimidyloxycarbonyl)-3', 6'-O, O'-diacetylfluorescein (CFSE), RNase A, RPMI-1640 and fetal bovine serum (FBS) were obtained from Invitrogen (Carlsbad, CA, USA). WST-1 assay kit was obtained from Roche (Penzberg, Germany). Fluorescence-labeled monoclonal antibodies against CD3 (FITC), CD69 (PE), CD25 (PE), TNF-α (PE), IFN-γ (APC), and IL-6 (PE) were obtained from BioLegend (San Diego, CA, USA). The primary antibodies for Western blotting and immunofluorescent staining were purchased from Cell Signaling Technology (Danvers, MA, USA). Annexin V-PE apoptosis detection kit and Cytometric Bead Array (CBA) mouse inflammation kit were obtained from BD Biosciences (San Jose, CA, USA).

### Isolation and culture of lymphocytes

Mice were sacrificed by cervical dislocation and the mesenteric lymph nodes were isolated. A single-cell suspension was prepared by passing the tissue through a 40-μm nylon cell strainer (BD Falcon). Cells were washed twice with phosphate-buffered saline (PBS), counted and resuspended in RPMI-1640 medium containing 10% FBS, penicillin 100 U/ml, streptomycin 100 µg/ml, 2 mM L-glutamine (Invitrogen) and 50 µM 2-mercaptoethanol (Invitrogen) (complete medium). Lymphocytes were seeded at a density of 2×10^6^ cells/ml in plates and incubated at 37°C in a humidified atmosphere of 5% CO_2_.

### Cell viability assay

Cell viability was measured using WST-1 assay kit (Roche, Penzberg, Germany) according to manufacturer’s instruction. The 50% inhibition concentration (IC_50_) indicates the concentration corresponding to 50% reduction of cell proliferation as compared with the positive control (Con A-activated cells).

### CFSE labeling assay

CFSE-labeling was performed as described previously [15]. Briefly, lymphocytes at 1×10^7^/ml were stained with CFSE (1 µM) for 10 min at 37°C, washed twice with PBS containing 10% FBS, and resuspended in complete medium. Lymphocytes at 2×10^6^/ml were seeded in a 96-well plate (100 µl/well) and treated with different concentrations of CuIIb for 1 h followed by stimulation with Con A (5 µg/ml) for 48 h. Cells were harvested and the fluorescence intensity of CFSE-labeled cells, which is decreased by one half with each cell division, was determined on a flow cytometer (FACSCalibur; Becton Dickinson, Mountain View, CA, USA), and the data were analyzed using the ModFit software (Verity Software House, Topsham, ME, USA).

### Analysis of CD69 and CD25 surface expression

Lymphocytes were seeded into a 24-well plate at 1×10^6^ cells per well in 500 µl of complete medium and stimulated with Con A (5 µg/ml) in the presence or absence of graded doses of CuIIb. After 24-h incubation at 37°C, cells were harvested and washed twice with PBS-F (PBS containing 0.1% NaN_3_ and 1% FBS), then stained with FITC-conjugated anti-CD3 and PE-conjugated anti-CD69 or PE-conjugated anti-CD25 monoclonal antibodies for 20 min. After washing with PBS-F, cells were fixed with 4% paraformaldehyde in PBS and then analyzed on a flow cytometer (FACSCalibur).

### Intracellular cytokine staining

Lymphocytes were pretreated with or without CuIIb at 37°C for 1 h. Then cells were co-incubated with PDB (0.1 µM), ionomycin (0.5 µg/ml) and monensin (2 µM) for another 4 h. Then, the cells were collected and stained with the FITC-conjugated monoclonal anti-CD3 antibody. After washing twice with PBS-F, the cells were fixed with 4% paraformaldehyde in PBS for 20 min at 4°C and subsequently washed with PBS-F, permeabilized with 0.1% saponin in PBS-F for 10 min at room temperature, and stained with anti-TNF-α-PE and anti-IL-6-PE for 20 min at 4°C. Samples were then analyzed on a flow cytometer (FACSCalibur).

### Determination of soluble cytokines

Lymphocytes were incubated in the presence or absence of CuIIb at 37°C for 1 h followed by stimulation with Con A (5 µg/ml) for 6, 24, and 48 h, respectively. Cytokines in the culture medium were quantitatively measured by CBA kit according to the manufacturer’s instruction. Data were acquired using CELLQuest software on a flow cytometer (FACSCalibur).

### Cell cycle analysis

Analysis of cell cycle was performed as described previously [8]. In brief, cells were fixed with 70% ethanol and stained with PBS containing 50 µg/ml propidium iodide (PI) and 30 µg/ml RNase A. DNA content data were acquired using CELLQuest software on a flow cytometer (FACSCalibur). A minimum of 20,000 events was collected for each sample.

### Immunofluorescence microscopy

Immunofluorescence analysis was performed as previously described [16]. Cells were fixed in 4% paraformaldehyde, permeabilized with ice-cold 100% methanol, and immunostained with mouse anti-β-actin and rabbit anti-p65 antibodies followed by CF488-conjugated goat-anti-mouse IgG and CF568-conjugated goat-anti-rabbit IgG, highly cross-absorbed (Biotium, Hayward, CA). Nuclei were revealed by Hoechst 33342 staining. Fluorescence images were collected under a Leica DMIRB fluorescent microscope (Leica Microsystems, Wetzlar, Germany) armed with a Spinning Disk Confocal Microscopy system (UltraView cooled CCD; Perkin Elmer, Waltham, MA, USA).

### Quantitative PCR

Real-time quantitative PCR (qPCR) was performed on Roche’s LightCycler 480 real-time PCR system. qPCR for each sample was performed twice in triplicates using a 20-µl reaction system containing 50 ng initial total RNA. Both annealing and extending temperature were set to 60°C. Forty PCR cycles were run and the melting curves were recorded. The primers used are as follows: 5′-TGAAGGACGAGGAGTACGAGC-3′ (sense) and, 5′-TGCAGGAACGAGTCTCCGT-3′ (anti-sense) for *IκBα*; 5′-CGTGGAACTGGCAGAAGAG-3′ (sense) and 5′-TGAGAAGAGGCTGAGACATAGG-3′ (anti-sense) for *TNF-α*; 5′- AGATCTGGCACCACACCTTCT-3′ (sense) and 5′-CTTTGATGTCACGCACGATTT-3′ (anti-sense) for *β-actin*. All other parameters for the reaction system and the PCR program were set according to the manufacturer’s protocol for the SYBR PrimeScript RT-PCR kit (Takara Bio, Dalian, China).

### Annexin V/7-AAD staining

After appropriate incubation, lymphocytes were collected and rinsed twice with cold PBS, resuspended in binding buffer. The cells were stained with PE-labeled Annexin-V/7-AAD for 15 min in the dark at room temperature. Apoptotic cells were analyzed on a flow cytometer (FACSCalibur).

### Western blotting

Western blot analysis was performed essentially as described previously [17]. In brief, whole cell lysates were obtained from lymphocytes stimulated with Con A (5 µg/ml) in the presence or absence of CuIIb. Immunoblotting was performed using antibodies against p-cofilin(S3), cofilin, p27^Kip1^, cyclin B1, cyclin D1, cyclin E, p-Erk1/2(T202/Y204), Erk1/2, p-JNK(T183/Y185), JNK, p38MAPK(T180/Y182), p38MAPK, p-NF-κBp65(S536), p65, p-IκBα(S32), IκBα, p-STAT3(Y705), STAT3, cleaved caspase-3, PARP, and β-tubulin. The densitometry of each band was quantified by FluorChem 8000 (AlphaInnotech; San Leandro, CA, USA).

### Statistical analysis

All experiments were performed at least three times, with one representative experiment shown. Data were presented as the mean ± standard deviation (SD). Statistical analysis was performed using GraphPad Prism 4.0 (GraphPad Software Inc., San Diego, CA, USA). One-way ANOVA, followed by the Newman–Keuls post-hoc test was used to compare between groups and a *P*-value <0.05 was considered as significant.

## Results

### Inhibitory effect of CuIIb on the cell proliferation of activated lymphocytes

We initially determined the acute cytotoxic effect of graded concentrations of CuIIb on mouse primary lymphocytes by using a modified MTT (WST-1) assay. The lymphocytes isolated from the lymph node contained mostly T, B and natural killer cells. We used these unsorted lymphocytes stimulated with Con A as a model of *in vitro* lymphocyte responses, which may mimic the *in vivo* system to some extent. The results showed that CuIIb of less than 11 µM had minimal cytotoxicity on the lymphocytes after 4-h treatment (Figure 1B). We then tested whether CuIIb suppressed the viability of these activated lymphocytes. As shown in Figure 1C and 1D, CuIIb dose-dependently inhibited the cell viability of Con A-stimulated lymphocytes with IC_50_ values of 4.05 ± 0.20 µM and 3.50 ± 0.25 µM for 24 h and 48 h, respectively. In accordance with the results of WST-1 assay, CFSE staining showed a similar inhibitory effect of CuIIb (2.5, 5 and 10 µM) on Con A-stimulated lymphocyte division (Figure 1E and 1F). Thus, three doses of CuIIb (2.5, 5 and 10 µM) without acute cytotoxicity were adopted for most of the following experiments.

### Cell cycle arrest in activated lymphocytes upon CuIIb treatment

**Figure 2:**
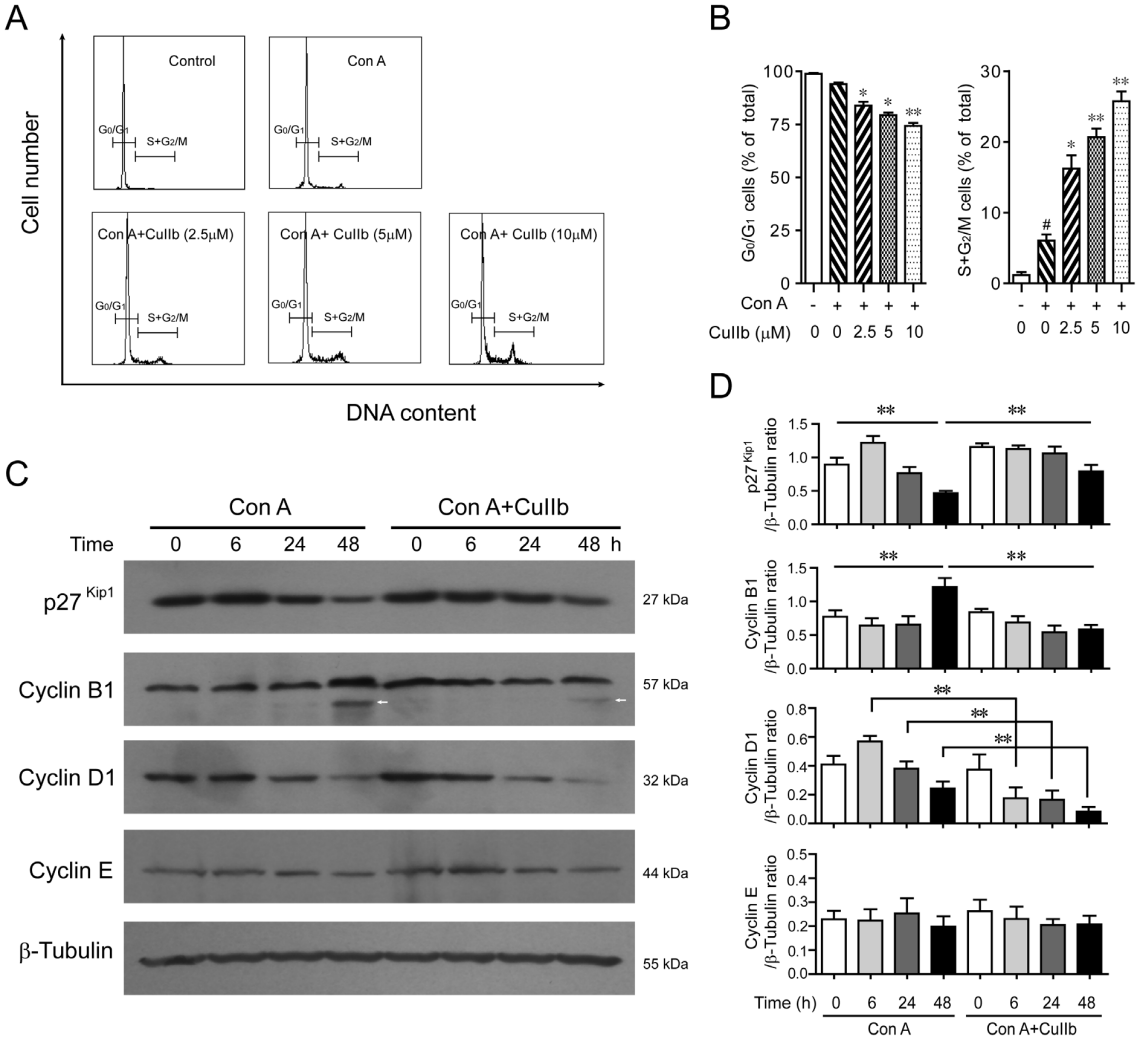
CuIIb induced cell cycle arrest by modulating the expression of cell cycle-related proteins in activated lymphocytes. (A and B) Flow cytometry analysis showing cell cycle distribution of Con A-stimulated lymphocytes upon CuIIb treatment for 48 h. #*P*<0.05 versus control group; *P<0.05 and **P<0.01 versus Con A group. (C and D) Cells were pretreated with CuIIb (10 µM) for 1 h, then exposed to Con A (5 µg/ml) for 0, 6, 24, and 48 h, respectively. The expression of p27^Kip1^ and cyclin proteins at various time points was determined by Western blotting. β-Tubulin was used as a loading control. Representative blots of three independent experiments are shown in (C) and the relative densitometric ratios of each protein to β-tubulin are shown in (D). Values are shown as mean ± SD of three experiments. Arrow indicates a nonspecific band. **P<0.01.

Given that CuIIb had affected cell cycle progression in prostate cancer cells [18], we next tested whether it could induce cell cycle arrest in Con A-stimulated lymphocytes. Cell cycle analysis using PI staining followed by flow cytometry showed that the resting lymphocytes (control) had a small proportion of cells in S+G_2_/M phases (1.15 ± 0.25%) (Figure 2A and 2B), while Con A stimulation significantly increased the S+G_2_/M cell proportion (6.05± 0.85%). CuIIb treatment further increased the proportion of S+G_2_/M cells in Con A-stimulated lymphocytes (up to 25.75 ± 1.35%), with G_0_/G_1_ cells being reduced (Figure 2A and 2B). This result suggested that CuIIb treatment arrested Con A-activated lymphocytes in S and G_2_/M phases, in line with its inhibitory effect on cell proliferation as determined by WST-1 and CFSE staining assay (Figure 1C-F).

As cell cycle arrest is usually associated with the modulation of cyclin-dependent kinase (CDK) inhibitors including p27^Kip1^ as well as cyclins [19], [20], we next examined the effect of CuIIb on p27^Kip1^ and cyclin expression in activated lymphocytes. Western blot analysis revealed that 10 µM CuIIb treatment for 48 h upregulated p27^Kip1^ expression in Con A-activated cells (Figure 2C and 2D), correlating with G_2_/M phase arrest. Consistent with this, cyclins B1 and D1 were modestly downregulated by CuIIb in Con A-activated cells (Figure 2C). In addition, CuIIb treatment had minimal effect on cyclin E expression. These results suggested that upregulation of p27^Kip1^ and downregulation of cyclins B1 and D1 may be involved in CuIIb-mediated cell cycle arrest in Con A-activated lymphocytes.

### Suppression of T lymphocyte activation by CuIIb treatment

**Figure 3:**
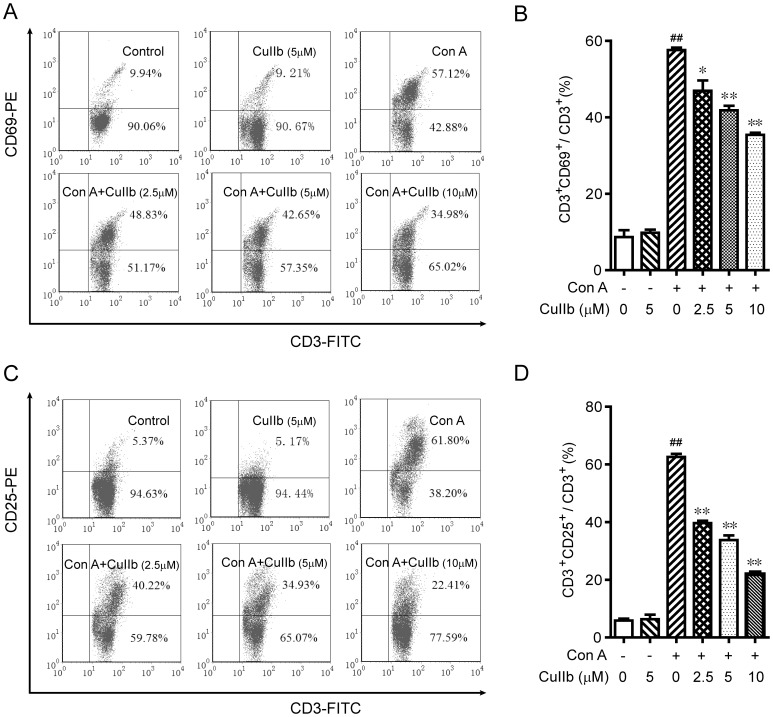
Inhibitory effect of CuIIb on CD69 and CD25 expression in activated lymphocytes. Mouse lymphocytes were stimulated with Con A in the presence or absence of CuIIb for 24 h. CD69 and CD25 expression was evaluated by flow cytometry. (A and C) Representative flow cytometric dot plots from one of three independent experiments. (B and D) Quantitative histograms of (A) and (C), respectively. Values are shown as mean ± SD of three experiments. ##*P*<0.01 versus control group; *P<0.05 and **P<0.01 versus Con A group.

Considering that Con A is more effective in activating T lymphocytes than other cells and that approximately 70−80% of the cells isolated from the lymph node were CD3^+^ T cells (data not shown), we sought to assess the effect of CuIIb on T cell activation in response to Con A stimulation. Flow cytometric analysis of the early activation marker CD69 and middle activation marker CD25 were used to monitor the activation status of T cells. As expected, CD69 and CD25 expression on CD3^+^ T cells was markedly upregulated by Con A stimulation (Figure 3A-D). Interestingly, CuIIb dose-dependently suppressed the expression of these activation markers on the T cells. These data indicated that Con A-induced T cell activation was significantly attenuated by CuIIb treatment.

### Inhibitory effect of CuIIb on inflammatory cytokine production

**Figure 4:**
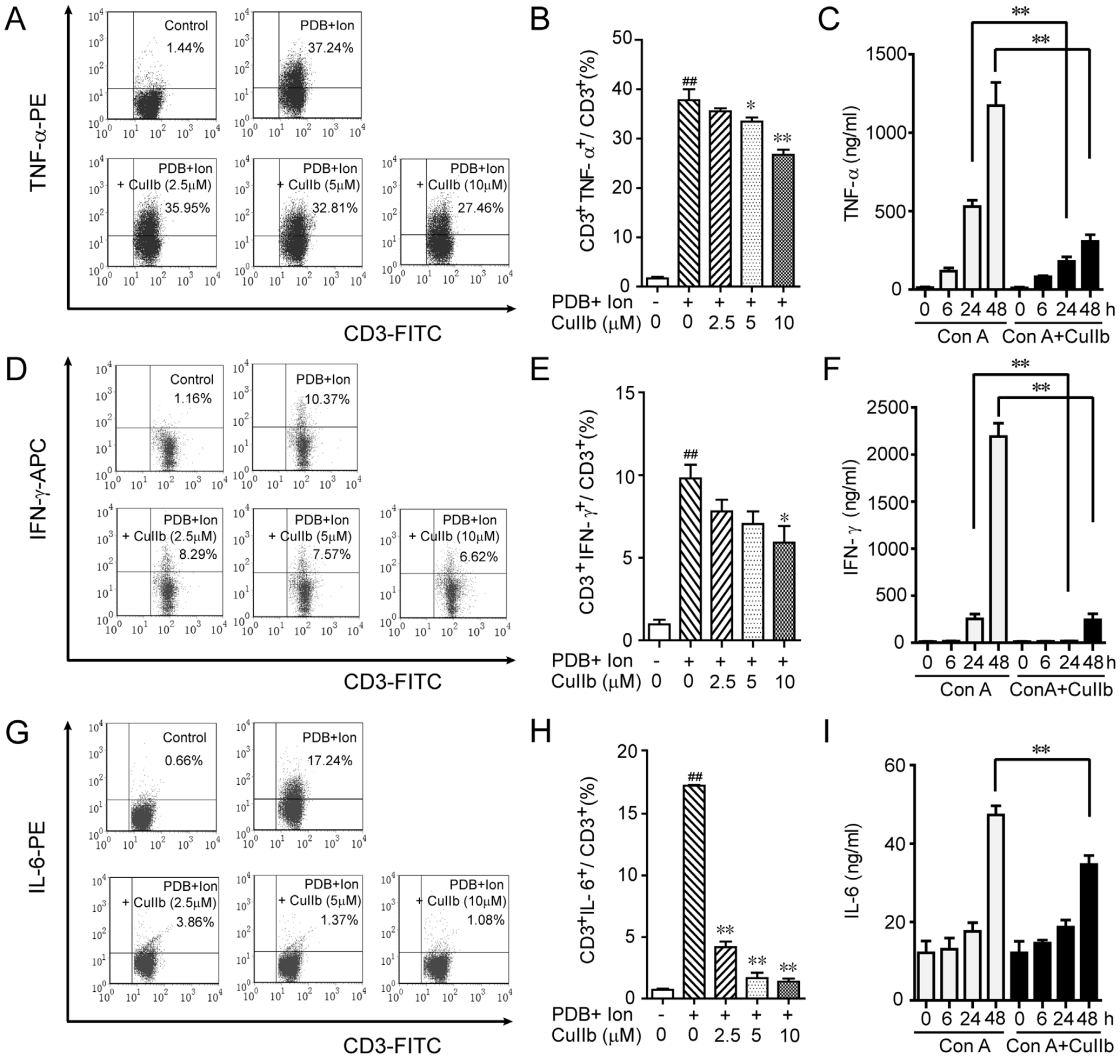
CuIIb suppressed inflammatory cytokine expression in activated lymphocytes. (A, D and G) Cells were pretreated with 2.5, 5 and 10 µM CuIIb for 1 h followed by PDB plus ionomycin (Ion) stimulation for 4 h, TNF-α, IFN-γ, and IL-6 were determined by intracellular cytokine staining. Both dot plots (A, D and G) and quantitative histograms (B, E and H) are presented to show the percentages of CD3^+^ T lymphocytes expressing TNF-α, IFN-γ or IL-6, respectively. (C, F and I) Lymphocytes were pretreated with 10 µM CuIIb for 1 h followed by Con A (5 µg/mL) stimulation for 0, 6, 24 and 48 h, TNF-α, IFN-γ, and IL-6 protein expression in culture medium were determined by cytometric bead array. Values are shown as mean ± SD (n = 3). ##*P*<0.01 versus control group; *P<0.05 and **P<0.01 versus PDB+Ion or Con A-activated group.

T cell-derived inflammatory cytokines, including TNF-α, IFN-γ and IL-6, play important roles in both the innate and adaptive immune responses as well as the development of inflammation-related diseases, and inhibiting their production may prevent the progression of inflammatory diseases. We thus tested the effect of CuIIb on inflammatory cytokine expression in T cells in response to PDB+Ion or Con A stimulation. Intracellular cytokine staining showed that the expression of TNF-α (Figure 4A and 4B), IFN-γ (Figure 4D and 4E) and IL-6 proteins (Figure 4G and 4H) in PDB+Ion-stimulated CD3^+^ T cells was significantly decreased upon CuIIb treatment. Consistent with this, cytometric bead array analysis of cytokines revealed that CuIIb remarkably reduced TNF-α, IFN-γ and IL-6 production in the culture medium of Con A-activated lymphocytes (Figure 4C, 4F and 4I). Together, these results indicated that CuIIb treatment could effectively suppress the inflammatory cytokine production in mitogen-activated T lymphocytes.

### Downregulation of MAPKs signaling by CuIIb in Con A-activated lymphocytes

**Figure 5:**
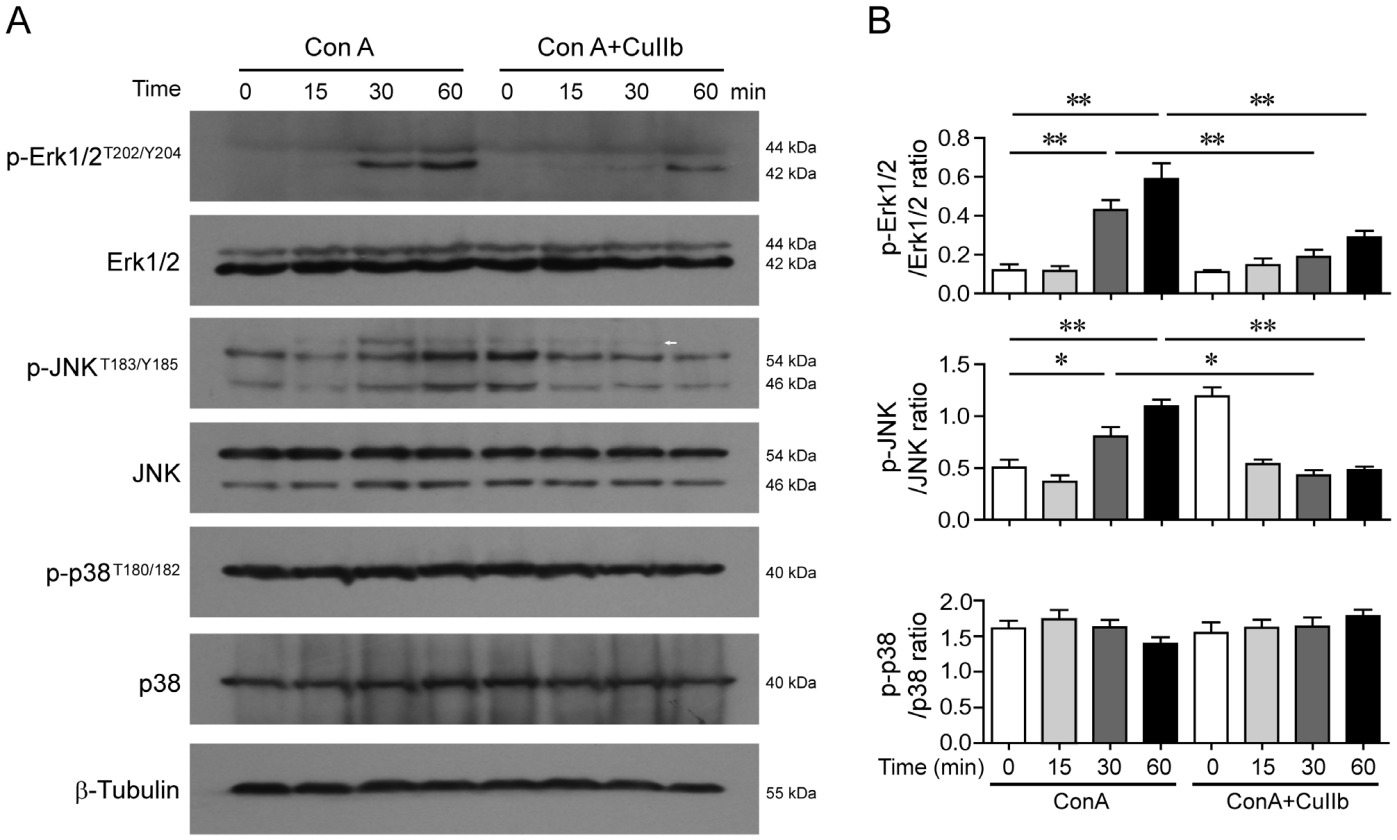
Effect of CuIIb on the activation of MAPKs in Con A-activated lymphocytes. Cells were pretreated with CuIIb (10 µM) for 1 h, and then exposed to Con A (5 µg/mL) for 0, 15, 30, and 60 min, respectively. The phosphorylation of Erk1/2, JNK and p38 MAPKs at various time points was determined by Western blotting. β-Tubulin was used as a loading control. Representative western blots are shown in (A) and the relative densitometric ratios of each protein to β-tubulin are shown in (B). Arrow indicates a nonspecific band. Values are shown as mean ± SD of three experiments. *P<0.05; **P<0.01.

As Con A stimulation of T lymphocytes mimics the T-cell receptor (TCR) engagement by its cognate antigens leading to T cell activation and cytokine production via upregulating multiple signaling pathways including MAPKs, we assessed whether CuIIb affected such signaling pathways in activated lymphocytes. Western blot analysis showed that CuIIb treatment markedly reduced Con A-induced phosphorylation of Erk1/2 and JNK (30 and 60 min) (Figure 5A and 5B), but there were no significant changes in p38 MAPK phosphorylation. In addition, short period pre-incubation with CuIIb could increase JNK phosphorylation in unstimulated cells (Con A + CuIIb, 0 h), consistent with the previous observed effect of cucurbitacin B [21]. These data indicated that the inhibitory effect of CuIIb on lymphocyte activation and inflammatory cytokine production was associated with the downregulation of Erk1/2 and JNK signaling.

### Modulation of NF-κB and STAT3 signaling in Con A-activated lymphocytes

**Figure 6:**
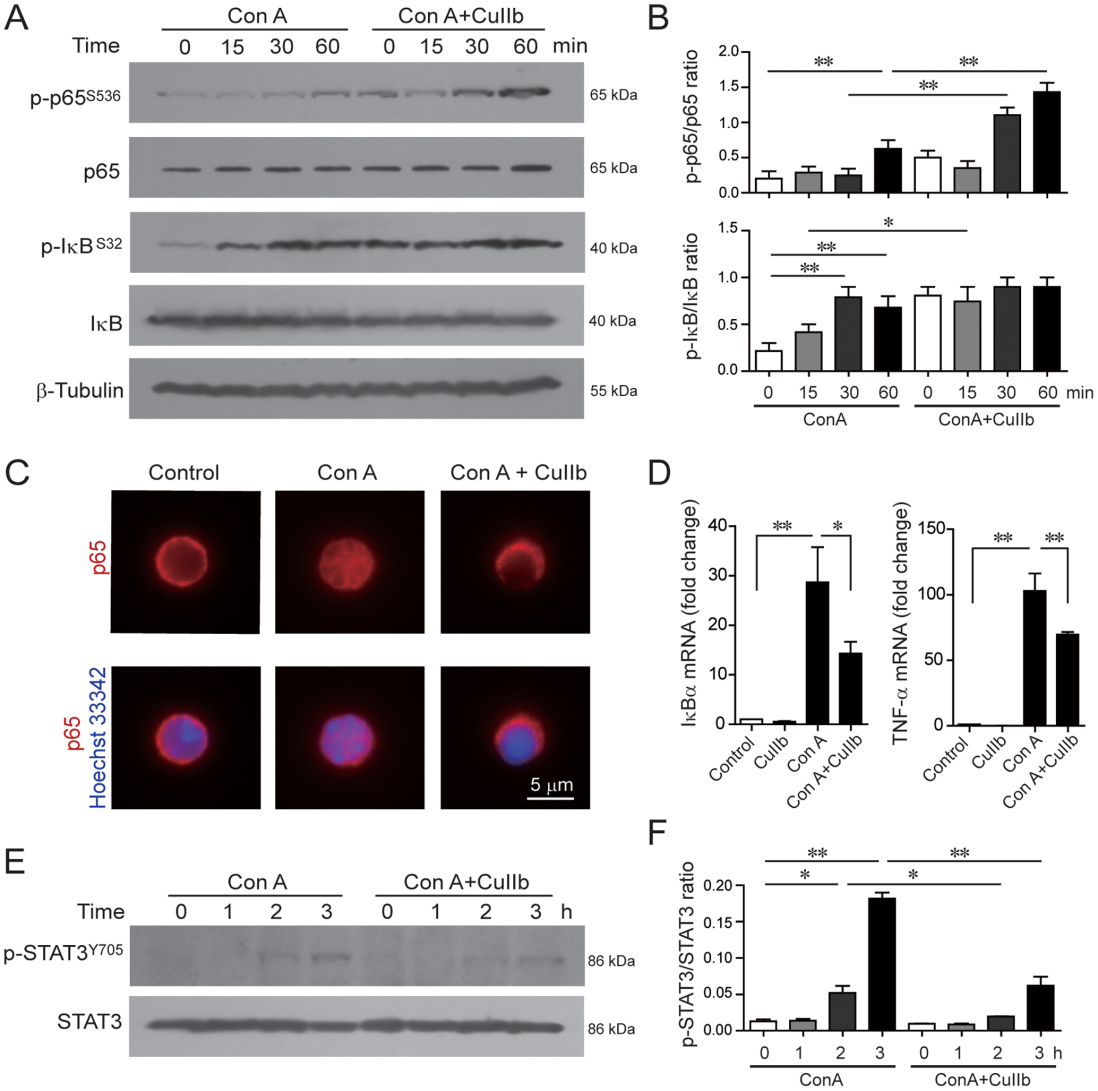
CuIIb modulated the NF-κB and STAT3 pathways in activated lymphocytes. Cells were pretreated with CuIIb (10 µM) for 1 h, then exposed to Con A for indicated time periods. The phosphorylation of NF-κB/p65, IκB (A) and STAT3 (E) at various time points was determined by Western blotting. β-Tubulin was used as a loading control. Representative western blots are shown in (A and E) and the relative densitometric ratios of each phosphorylated protein to its total protein are shown in (B and F). (C) Immunofluorescence analysis of the distribution of NF-κB/p65 in lymphocytes. After co-treatment with CuIIb and Con A for 1 h, cells were fixed and then immunostained with anti-p65 antibody (red) followed by CF568-labeled second antibody. Nuclei (blue) were revealed by Hochest 33342 staining. Fluorescent images were obtained by fluorescence microscopy with a 100× oil objective lens. Representative of at least 10 images for each group were shown. Scale bar, 5 µm. (D) Quantitative PCR analysis of the mRNA levels of *IκBα* and *TNF-α.* CuIIb were pretreated for 1 h prior to stimulation with Con A. Levels of *IκBα* and *TNF-α* mRNA were determined 3 h after Con A stimulation. The mRNA levels normalized to *β-actin* are expressed as mean ± SD with controls defined as 1 (n  =  3). *P<0.05; **P<0.01.

NF-κB is a crucial transcription factor that controls the expression of many genes involved in immune responses, including cytokines *TNF-α*, *IFN-γ* and *IL-6*. To examine the effect of CuIIb on the NF-κB pathway in activated lymphocytes, we determined the phosphorylation of RelA/p65 and IκBα by Western blotting. As shown in (Figure 6A and 6B), there was a time-dependent increase in the phosphorylation levels of p65 and IκBα in response to Con A stimulation; surprisingly, CuIIb treatment slightly increased the phosphorylation level of IκBα after 15-min stimulation and markedly increased the phosphorylation levels of NF-κB at both 30-min and 60-min stimulation. Interestingly, immunofluorescence microscopy revealed that CuIIb treatment blocked the nuclear translocation of p65 in Con A-stimulated lymphocytes (Figure 6C). These results suggested that CuIIb inhibited Con A-induced NF-κB signaling by suppressing its nuclear translocation but not its phosphorylation.

As NF-κB is a transcription factor that regulate gene expression in the nucleus, we hypothesized that blocking its nuclear translocation might affect the transcription of its target genes. To verify this, we analyzed the transcription of NF-κB target genes *IκBα* and *TNF-α* [22], [23]. Quantitative PCR analysis showed that whereas CuIIb alone did not change the mRNA levels of *IκBα* and *TNF-α*, it significantly decreased Con A-induced upregulation of *IκBα* and *TNF-α* mRNA levels (Figure 6D).

Given that CuIIb markedly suppressed IL-6 production and that the Jak/STAT3 pathway is activated by IL-6 [24], we tested whether STAT3 phosphorylation is affected by CuIIb treatment. Western blot analysis revealed that there was a time-dependent increase in the phosphorylation levels of STAT3 in response to Con A stimulation, whereas CuIIb significantly inhibited its phosphorylation (Figure 6E and 6F).

### Disruption of actin dynamics by CuIIb in Con A-activated lymphocytes

**Figure 7:**
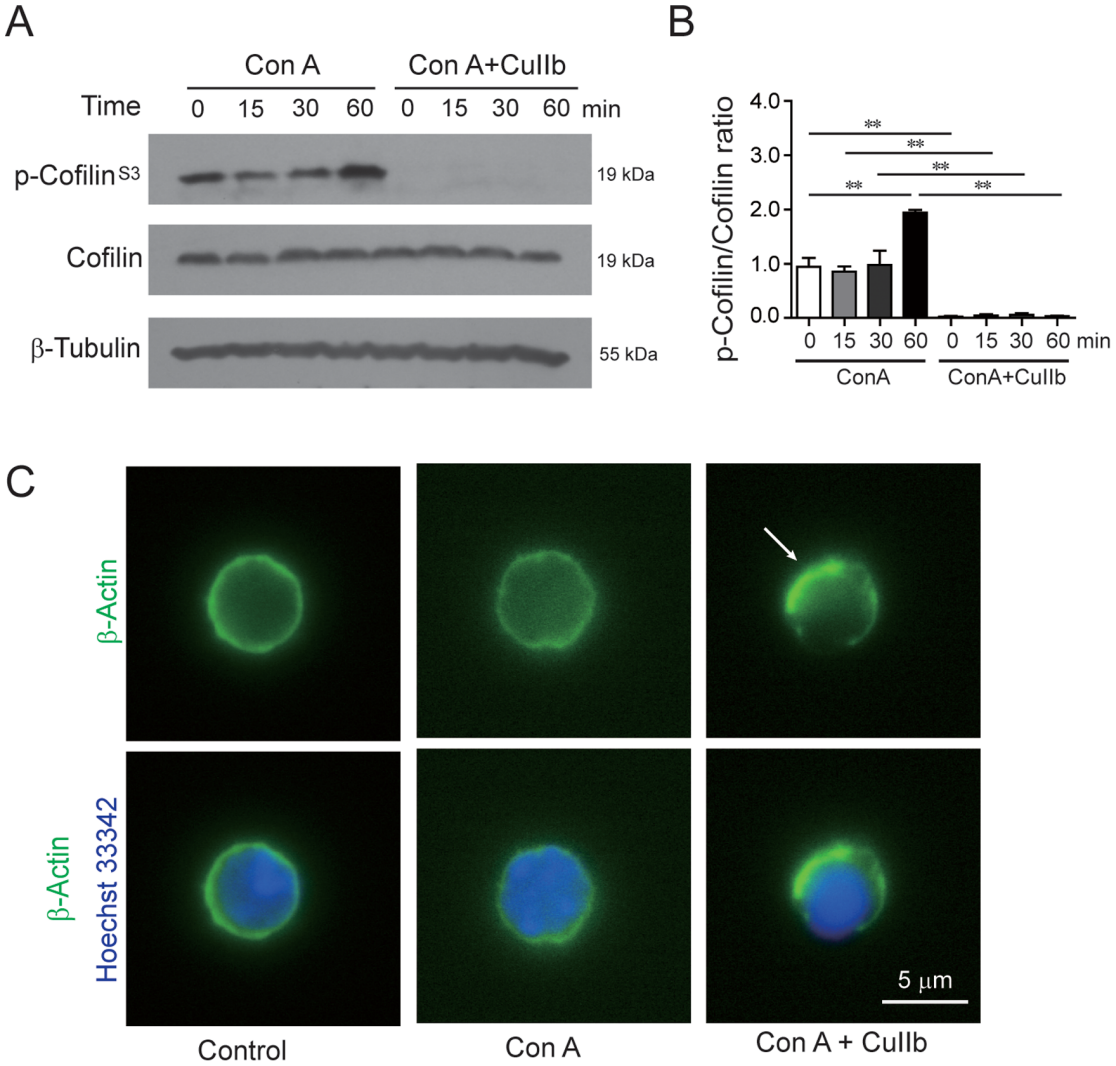
CuIIb disrupted actin dynamics in activated lymphocytes. Cells were pretreated with CuIIb (10 µM) for 1 h, then exposed to Con A for indicated time periods. The phosphorylation of cofilin at various time points was determined by Western blotting. β-Tubulin was used as a loading control. Representative blots are shown in (A) and the relative densitometric ratios of P-cofilin to cofilin are shown in (B). **P<0.01. (C) Immunofluorescence analysis of the distribution of β-actin in lymphocytes. After co-treatment with CuIIb and Con A for 1 h, cells were fixed and then immunostained with anti-β-actin antibody followed by appropriate second antibody. Nuclei (blue) were revealed by Hochest 33342 staining. Fluorescent images were obtained by fluorescence microscopy with a 100× oil objective lens. Representative of at least 10 images for each group were shown. Arrow indicates aggregated actin. Scale bar, 5 µm.

As CuIIb has previously been reported to damage the actin cytoskeleton in prostate cancer cells [18], we assessed its effect on the actin cytoskeleton in lymphocytes. Western blot analysis showed that cofilin, a critical regulator of actin dynamics, had increased phosphorylation after exposure to Con A at 60 min. However, most of the cofilin was rapidly de-phosphorylated in Con A-stimulated cells pre-treated with CuIIb (Figure 7A and 7B), indicating a disruption of actin dynamics required for normal cell cycle progression. Consistent with this, immunofluorescence analysis revealed that CuIIb treatment induced actin aggregation in Con A-activated lymphocytes (Figure 7C).

### Enhanced apoptosis in activated lymphocytes upon CuIIb treatment

**Figure 8:**
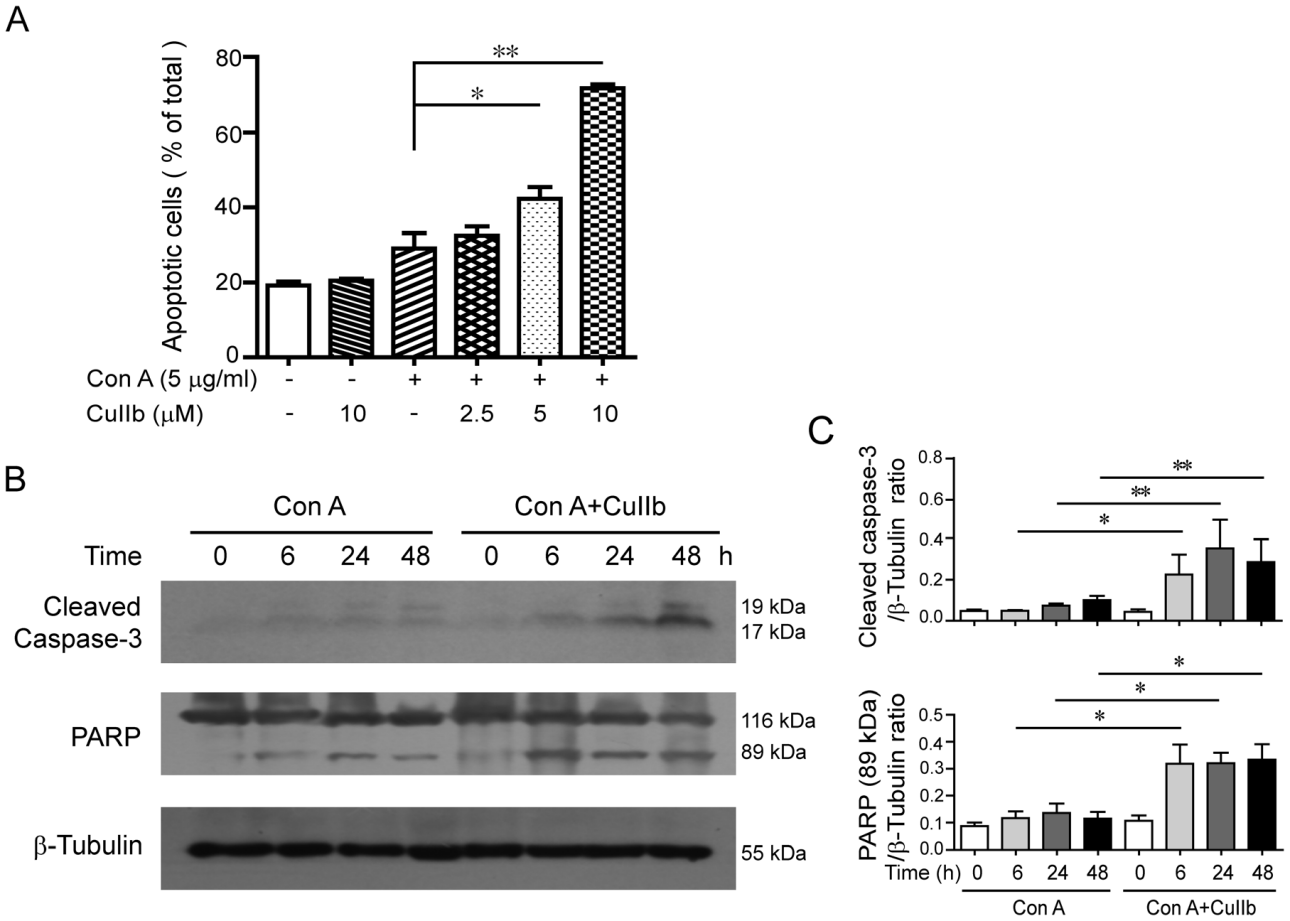
CuIIb-induced cell apoptosis in Con A-stimulated mouse lymphocytes. (A) Cells were stimulated with Con A in the absence or presence of CuIIb for 24 h. Apoptosis was determined by annexin V-PE/7-AAD staining. The percentages of annexin V^+^ cells (sum of 7-AAD^−^ annexin V^+^ cells and 7-AAD^+^annexin V^+^ cells) within total cells are shown. Values are shown as mean ± SD (n = 3). (B and C) Cells were pretreated with CuIIb (10 µM) for 1 h, then exposed to Con A for indicated time periods. The cleaved caspase-3 and PARP levels were determined by Western blotting. β-Tubulin was used as a loading control. Representative blots of three independent experiments are shown in (B) and the relative densitometric ratios of each protein to β-tubulin are shown in (C). *P<0.05; **P<0.01.

We finally assessed whether CuIIb treatment induced apoptotic cell death in Con A-activated lymphocytes. Annexin V staining analysis showed that there was a slight increase of apoptosis in Con A-stimulated cells as compared to control (Figure 8A). Co-treatment with CuIIb further increased apoptotic cell death in Con A-stimulated cells. This result is supported by the Western blot analysis showing that lymphocytes co-treated with Con A and CuIIb had increased levels of cleaved caspase-3 and fragmented poly (ADP-ribose) polymerase (PARP) (89 kDa) as compared to Con A alone (Figure 8B and 8C). These data indicated that CuIIb could enhance apoptosis in Con A-activated lymphocytes.

## Discussion

Lymphocytes play a central role in regulating both the innate and adaptive immune responses. To exhibit their functions, lymphocytes must undergo a series of cellular processes including activation, proliferation, differentiation and cytokine expression when encountering specific antigens [25]. Such processes of lymphocytes represent important targets for immunosuppressive or anti-inflammatory drugs [13]. In this study, we demonstrated that CuIIb robustly attenuated the activation and proliferation of mitogen-activated lymphocytes. Importantly, the critical inflammatory cytokines, including TNF-α, IFN-γ and IL-6, were markedly downregulated and the activation-induced cell death was enhanced by CuIIb treatment. These data highlight the novel anti-inflammatory activity of CuIIb in downregulating the functions of activated lymphocytes in inflammation-related diseases.

The activation of T lymphocytes is a critical process preceding clonal expansion upon antigen stimulation, and such a process is manifested by the surface expression of activation markers, such as CD69 and CD25. As an early activation marker, CD69 functions as a costimulatory molecule which enhances the T responses following the TCR-ligand interaction [26], [27]. Another activation marker CD25 is the α subunit of IL-2 receptor (IL-2R) [28], which comprises the high affinity IL-2R with the β and γ subunits. Thus, CD25 upregulation is required for IL-2-mediated signaling [29]. During adaptive immune responses, IL-2 serves as a critical growth factor for lymphocytes, which binds the high affinity IL-2R and drives the activated lymphocytes into cell division, resulting in a robust clonal expansion [26]. We observed in this study that CuIIb strongly suppressed the expression of both CD69 and CD25, thus dampening the costimulatory and IL-2 signaling required for clonal expansion of lymphocytes. In line with this, there was a significant reduction in Con A-stimulated cell proliferation upon CuIIb treatment. As the expression of CD69 and CD25 was regulated by the MAPK pathways [29], CuIIb-triggered downregulation of Erk1/2 and JNK may at least partly be responsible for the decreased expression of such activation markers. Together, CuIIb-mediated downregulation of CD69 and CD25 expression contributed to its anti-proliferative effect on lymphocytes in response to mitogen stimulation.

Apart from downregulating CD69 and CD25 expression, CuIIb also induced cell cycle arrest in S and G_2_/M phases in activated lymphocytes, which is in agreement with the previous study that this same agent caused a similar cell cycle arrest in human prostate cancer cells [18]. Like its closely related analogue cucurbitacin IIa and other members of cucurbitacin family [4], [30], [31], CuIIb could rapidly disrupt the actin dynamics and damage the actin cytoskeleton leading to the formation of actin aggregates in lymphocytes. Given the essential role of actin dynamics in cell cycle progression, CuIIb-caused actin dynamics disruption would impair the normal lymphocyte division process. Furthermore, the actin cytoskeleton damage caused by CuIIb might activate the cell cycle checkpoint, as evidenced by the upregulation of p27^Kip1^ (a cyclin-dependent kinase inhibitor) and downregulation of cyclin B (an S-phase cyclin required for G_2_-M transition). Thus, it appeared that CuIIb induced cell cycle arrest by damaging the actin cytoskeleton, which may also contribute to its anti-proliferative effect in mitogen-activated lymphocytes.

Proinflammatory cytokines, including TNF-α, IFN-γ and IL-6, have an important role not only in immune responses against infection but also in pathological inflammation [32], [33], [34]. As a potent proinflammatory cytokine, TNF-α is released both by macrophages and T lymphocytes in response to bacterial infection [33]. It initiates inflammatory responses through stimulating secretion of other proinflammatory cytokines, such as IL-1β, IFN-γ and IL-6, leading to the production of secondary inflammatory mediators, including prostaglandins, leukotriene and nitric oxide [33]. Among these inflammatory mediators, IL-6 possesses a wide variety of activities, including the stimulation of mononuclear differentiation as well as the promotion of acute phase reaction protein expression [10], [35]. Similarly, IFN-γ acts on many innate immune cells including macrophages and triggers their functions [34]. We observed that upon CuIIb treatment, the production of TNF-α, IFN-γ and IL-6 were markedly decreased in either PDB+ionomycin- or Con A-activated T lymphocytes. Our data is consistent with one previous study showing that cucurbitacin R could suppress TNF-α production both in rats and in human lymphocytes [36]. Given the importance of these cytokines in inflammatory responses, our data suggested that CuIIb-induced downregulation of proinflammatory cytokines contributed to its therapeutic activity in controlling inflammatory diseases such as intestinal inflammation and acute tonsillitis, although additional *in vivo* research is warranted.

It has been well known that the transcription of inflammatory cytokines is coordinately regulated by the NF-κB and MAPK pathways [37]. As the NF-κB is the critical transcription factor that regulates the expression of proinflammatory cytokines [38], blocking NF-κB nuclear translocation by CuIIb might lead to decreased transcription of NF-κB target genes; this is evidenced by the reduced mRNA levels of *TNF-α* and *IκBα*, two such NF-κB target genes [22], [23]. In addition, NF-κB coordinates with JNK, Erk1/2 and p38 signaling for initiation of the inflammatory response, culminating in the expression of proinflammatory cytokines including TNF-α, IFN-γ and IL-6 [39], [40]. Therefore, CuIIb-induced suppression of Erk1/2 and JNK phosphorylation as well as the blockade of NF-κB nuclear translocation was responsible for suppressing the expression of inflammatory cytokines in activated lymphocytes.

One interesting finding of this study is that CuIIb could block NF-κB p65 nuclear translocation even though it enhanced its phosphorylation. Our data are in agreement with a previous study that cucurbitacin E blocks NF-κB(p65) nuclear translocation with no significant effect on its phosphorylation in LPS-stimulated RAW 264.7 cells [41]. As NF-κB nuclear transport is dependent upon the dynein molecular motor and normal actin cytoskeleton [42], [43], the disruption of the actin cytoskeleton by CuIIb might have a role in the blockade of NF-κB(p65) nuclear translocation. Particularly, CuIIb-induced cofilin dephosphorylation (i.e. activation) may be involved in preventing NF-κB(p65) nuclear translocation as one previous study showing that cofilin has an essential role in NF-κB(p65) nuclear translocation [44]. However, additional investigation is warranted to reveal the precise mechanism for cucurbitacin-induced blockade of NF-κB(p65) nuclear translocation.

Activation-induced cell death of T lymphocytes is a critical step for the immune system to dampen the inflammatory response after the clearance of infectious pathogens. It is a programmed cell death (apoptosis) that requires the activation of a series caspases including caspase-3 [45]. We observed an increase in apoptosis when the lymphocytes were activated with the mitogen Con A, which was further enhanced by CuIIb treatment. This result is in agreement with previous studies showing that cucurbitacin IIa and R induced apoptosis in lipopolysaccharide-stimulated RAW 264.7 cells [8], [46]. Given that activation-induced cell death plays a key role in dampening inflammation, the enhancement of apoptosis by CuIIb in activated lymphocytes may also contribute to its anti-inflammatory activity.

In summary, our results demonstrated that CuIIb inhibited the activation, proliferation, and proinflammatory cytokine expression in Con A-stimulated lymphocytes. Such inhibitory effects of CuIIb were probably mediated by blocking nuclear translocation of NF-κB, downregulating JNK, Erk1/2, and STAT3 signaling, and disrupting actin dynamics in activated lymphocytes. Our data suggest that CuIIb exhibits its therapeutic activity in inflammation-related diseases through modulating multiple cellular behaviors and signaling pathways leading to the suppression of adaptive immune responses.
